# Supplementary material for: A novel regulator of the fungal phosphate starvation response revealed by transcriptional profiling and DNA affinity purification sequencing
Source: mBio. 2025 Aug 25;16(10):e02023-25. doi: 10.1128/mbio.02023-25 (PMC12506109; doi:10.1128/mbio.02023-25)
Supplement: Supplemental material — s and methods, figures, and tables. [file mbio.02023-25-s0004.pdf]

A novel regulator of the fungal phosphate starvation response revealed by transcriptional profiling and DNA affinity purification sequencing

Lori B. Huberman, Vincent W. Wu, David J. Kowbel, Juna Lee, Chris Daum, Vasanth R. Singan, Igor V. Grigoriev, Ronan C. O'Malley, and N. Louise Glass

Supplemental Datasets Legends

Dataset S1. TPM counts and differential expression analysis of wild type, Δ*nuc-1*, and Δ*nuc-3* cells exposed to the indicated concentration of phosphate.

Dataset S2. DAPseq data for NUC-1 and NUC-3.

Dataset S3. RT-qPCR data from wild type and mutant cells exposed to phosphate starvation.

Supplemental Materials and Methods, Tables, and Figures

RNA sequencing and transcript abundance

The indicated strains were inoculated into 3 mL Vogel's minimal medium (1) in 24-well plates at 10^6 conidia/mL and grown for 24 h at 25°C in constant light with constant shaking at 200 rpm. The media was then vacuumed out of the wells and mycelial mats were washed three times with either 3 mL Fries minimal medium (2, 3) containing 7 mM phosphate or Fries minimal medium lacking phosphate and then shifted to 3 mL of either Fries minimal medium containing 7 mM phosphate or Fries minimal medium lacking phosphate, respectively. Cells were then incubated for the indicated amount of time at 25°C in constant light with constant shaking at 200 rpm.

Mycelia were harvested by filtering on Whatman paper no. 1 and flash freezing in liquid nitrogen. RNA extraction and library preparation were performed as described in Wu *et al* (2020) (4). Briefly, total RNA was harvested from mycelia flash frozen in liquid nitrogen the indicated amount of time post-transfer to the indicated media using a TRIzol (Life Technologies) extraction and cleaned up with the RNeasy kit (Qiagen). Libraries were prepared from total RNA using poly(A) enrichment and standard Illumina protocols. Libraries were prepared and sequenced at the Joint Genome Institute on the Illumina HiSeq platform with 100 bp single end reads or at the University of California Berkeley Vincent J. Coates Genomics Sequencing Lab on the Illumina HiSeq platform with 50bp single end reads or NovaSeq platform with 150 bp paired end reads as listed in Table S2.

The transcript abundance (transcripts per million, TPM) was quantified using Salmon v. 1.4.0 mapping to the *N. crassa* OR74A genome (v12) (5) with the --validateMappings setting (6). Differential expression was determined using DESeq2 v. 1.44.0 (7). Differential expression was only called between samples sequenced at the same center and sequencing platform. Genes were denoted as differentially expressed between wild type exposed to Fries minimal medium with 7 mM phosphate as compared to Fries minimal medium lacking phosphate and wild type (*nuc-1*$^+$) cells as

compared to Δ*nuc-1* cells exposed to Fries minimal medium lacking phosphate if there was at least a 4-fold change in expression and the TPM of the gene was at least 10 in either of the 2 conditions. Genes were denoted as differentially expressed between wild type (*nuc-3*$^{+}$) cells as compared to Δ*nuc-3* cells exposed to Fries minimal medium lacking phosphate if there was at least a 2-fold change in expression and the TPM of the gene was at least 10 in either of the 2 conditions.

RNAseq data used in this study were deposited in the Gene Expression Omnibus (GEO) at the National Center for Biotechnology Information (NCBI) and are accessible through GEO series accession number GSE293601. Processed RNAseq data are available in Dataset S1.

DAPseq

DAPseq was performed as described in (4). Briefly, cDNA was generated from RNA harvested from the wild type FGSC 2489 using the EcoDry premix (Clontech). Predicted open reading frames for *nuc-1* (NCU09315) and *nuc-3* (NCU03077) were amplified and inserted into an expression vector upstream of a HALO tag. NUC-1 and NUC-3 proteins were then produced using the Promega TnT T7 Rabbit Reticulocyte Quick Coupled Transcription/Translation System by incubating 1 μg of plasmid DNA with 60 μL of TnT Master Mix and 1.5 μL of 1 mM methionine overnight at room temperature.

The NUC-1 and NUC-3 transcription and translation reactions were incubated with 1 μg salmon sperm for blocking, 20 μl Promega Magne HaloTag Beads, and 20 ng of genomic DNA libraries prepared with the KAPA library kit for Illumina sequencing from genomic DNA harvested from the wild type FGSC 2489 strain grown on liquid Vogel's minimal medium for 24 h at 25°C, sheared to a 300 bp peak using a Covaris LE220 sonicator, and size selected using AMPure XP beads on a rotator for 1 h at room temperature. The bead-bound protein and protein-bound DNA were then washed three times with 2.5% Tween20 in phosphate buffered saline, resuspended in 30 μL ddH2O, and heated to 98°C for 10 m to denature proteins and release bound DNA into solution. The supernatant containing any released DNA was transferred to a new tube for PCR amplification using KAPA HiFi polymerase for 12-16 cycles to generate DAPseq DNA libraries. A DAPseq DNA library was generated in the same conditions with no plasmid added to the TnT Master Mix as a negative control. Single DAPseq libraries were generated for NUC-1 and NUC-3 and sequenced with 150 bp paired end reads on an Illumina MiSeq.

Filtered reads were aligned to the *N. crassa* OR74A genome (v12) using Bowtie v2.3.2 (8). Peak calling was performed using MACS v2.1.1 with p-value cutoff at 0.001 and utilizing negative control library alignments (9). Peaks within 3,000 bp upstream of a translation start site were called as associated with that gene. DAPseq data was deposited in the NCBI Sequence Read Archive (accession number PRJNA436200; ID SRP133627).

DNA binding consensus motif generation

Motif discovery was performed using Multiple Expectation maximizations for Motif Elicitation (MEME) v5.5.5 (NUC-1) or v5.5.7 (NUC-3) (10). The input for MEME motif discovery was DAPseq binding peak sequences with a maximum motif width of 50 bp, a

minimum motif width of 5 bp, any number of motif sites in the sequences, the classic objective function, and a 0th order Markov model for sequences. Binding peak sequences were included in motif generation for NUC-1 or NUC-3 if they were within 3,000 bp upstream of a translational start site that was at least 2-fold differentially expressed between wild type and either the Δ*nuc-1* or Δ*nuc-3* mutant, respectively, during exposure to Fries minimal medium lacking phosphate and had a TPM of at least 10 in either wild type or the Δ*nuc-1* or Δ*nuc-3* mutant, respectively.

Functional enrichment analysis and gene annotation

Functional enrichment analysis was done using the FungiFun2 online resource tool with KEGG as the classification ontology (11, 12). The gene to category associations were tested for overrepresentation using hypergeometric distribution with Benjamini-Hochberg correction for false discovery rate.

Gene annotations were pulled from FungiDB (https://fungidb.org) (13) or inferred from homology to characterized genes in related fungi.

NUC-3 phylogenetic tree generation and protein alignment

The Basic Local Alignment Search Tool for proteins (BLASTP) was used to identify NUC-3 homologs using the NCBI reference proteins database of the indicated species and the following settings: blastp algorithm, 100 max target sequences, short queries, 0.05 expect threshold, a word size of 4, 0 max matches in query range, the BLOSUM62 matrix, gap costs of existence 11 and extension 1, the conditional compositional score matrix adjustment, and filtering low complexity regions.

Identified homologs were aligned with MAFFT v7.487 using the FFT-NS-2 strategy (14). The phylogenetic tree was constructed using FastTree v2.1.8 using the maximum likelihood method based on the Jones-Taylor-Thornton matrix-based model with *Candida albicans* Try6 as the outgroup (15). The reliability of each split in the tree was computed using the Shimodaira-Hasegawa test on three alternate topologies around that split.

The alignment of the basic helix-loop-helix domains of NUC-1 and NUC-3 homologs was performed with MAFFT v7.490 using the FFT-NS-2 strategy (14). The basic helix-loop-helix domain annotation (amino acids 635-745 for NUC-1 and amino acids 154-204 for NUC-3) was taken from UniProt (16). The pairwise sequence alignment of NUC-1 and NUC-3 was performed using Lalign (17, 18) (https://www.ebi.ac.uk/jdispatcher/psa/lalign) using the standard settings. AlphaFold structural predictions of NUC-1 and NUC-3 were retrieved from the AlphaFold DB version 2022-11-01, created with the AlphaFold Monomer v2.0 pipeline (19, 20). The pairwise structural alignment of NUC-1 and NUC-3 was performed using the AlphaFold predicted structures of NUC-1 and NUC-3 (19) in the RCSB protein data bank using the TM-align method (21).

SI FIGURES

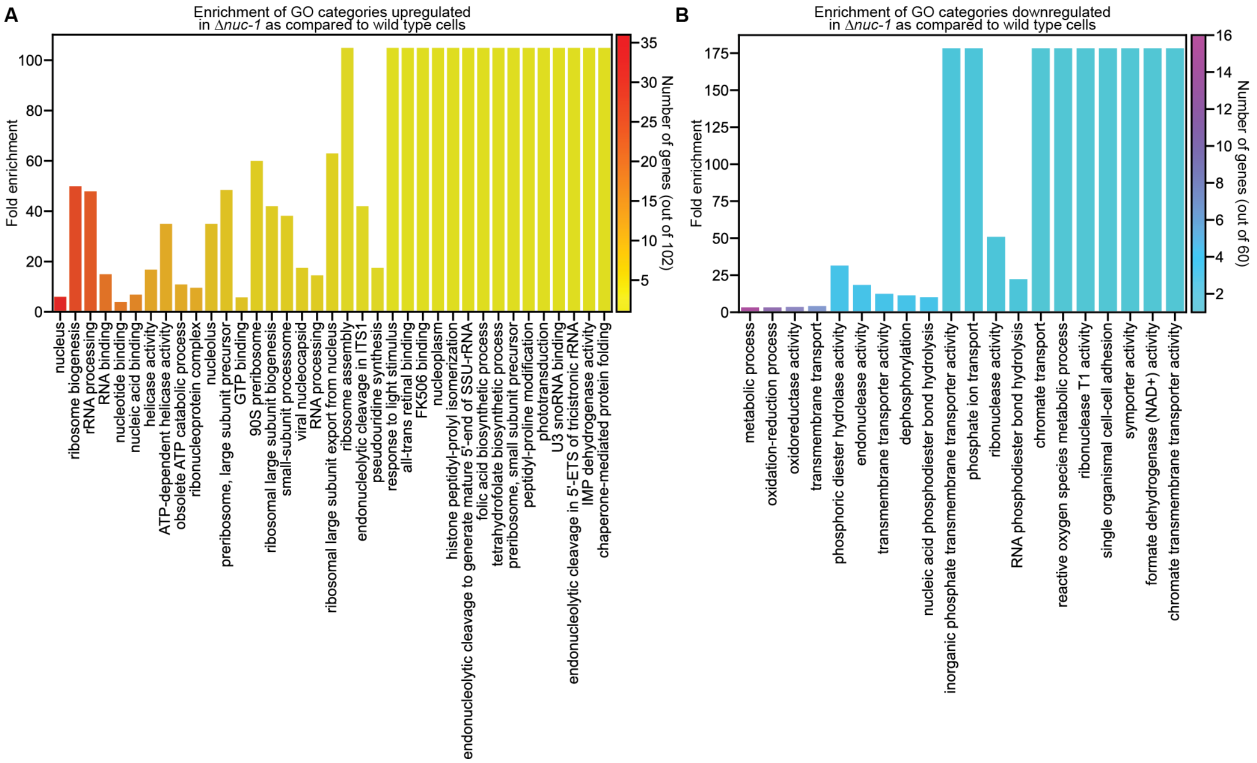

Fig S1. The transcription factor NUC-1 activates genes involved in phosphate acquisition and liberation and represses genes associated with the ribosome and transcription. (A) Fold enrichment of genes significantly upregulated by at least 4-fold in the Δ*nuc-1* mutant as compared to wild type (*nuc-1*$^{+}$) cells during phosphate starvation in significantly enriched ($p_{adj} < 0.05$) gene ontology (GO) categories. The number of genes significantly upregulated by at least 4-fold found in each category is indicated by the color of the bar. **(B)** Fold enrichment of genes significantly downregulated by at least 4-fold in the Δ*nuc-1* mutant as compared to wild type (*nuc-1*$^{+}$) cells during phosphate starvation in significantly enriched ($p_{adj} < 0.05$) gene GO categories. The number of genes significantly downregulated by at least 4-fold found in each category is indicated by the color of the bar. GO enrichment analysis was calculated using FungiFun 2.2.8 (11).

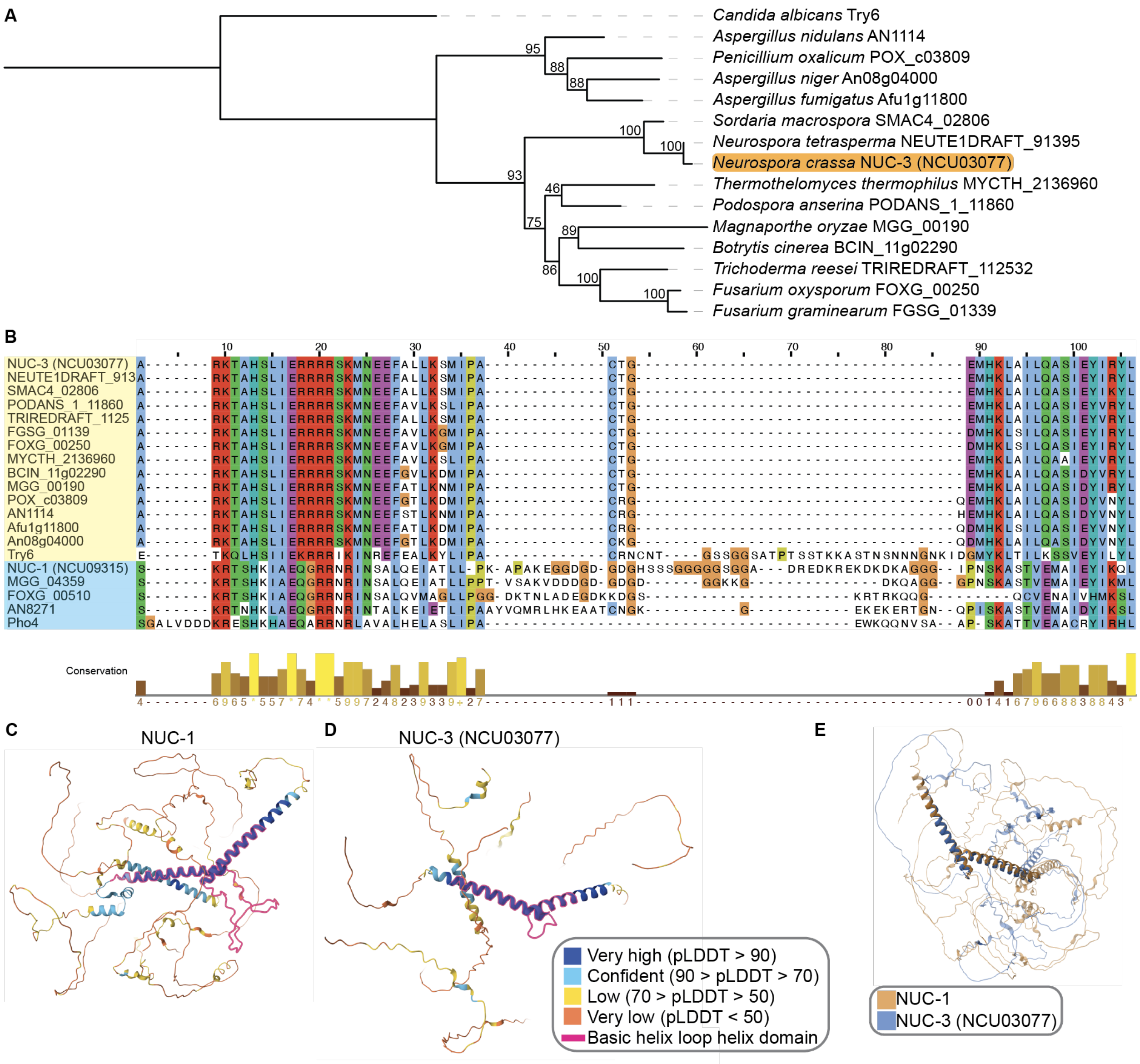

Fig S2. NUC-3 is conserved in the Pezizomycotina. (A) Protein sequences of NUC-3 homologs were used to build a phylogenetic tree using the maximum likelihood method based on the Jones-Taylor-Thornton matrix-based model using FastTree with *Candida albicans* as an outgroup (15). The reliability of each split in the tree was computed using the Shimodaira-Hasegawa test on three alternate topologies around that split. *N. crassa* NUC-3 is highlighted in orange. Numbers at each node indicate the bootstrap support for the branch. **(B)** Alignment of the basic helix-loop-helix domains (as annotated by UniProt (16)) of NUC-3 and NUC-1 homologs (amino acids 635-745 for NUC-1 and amino acids 154-204 for NUC-3). The NUC-3 homologs (highlighted in yellow) are the same proteins as in **(A)**. The NUC-1 homologs (highlighted in blue) are: *Magnaporthe oryzae* MGG_04359, *Fusarium oxysporum* FOXG_00250, *Aspergillus nidulans* AN8271, and *Saccharomyces cerevisiae* Pho4. Protein sequences were aligned using MAFFT (14). Colors of amino acids are determined using the Clustal color scheme.

Conservation is determined using the AMAS method (22) in Jalview (23) and is a numerical index reflecting conservation of the physico-chemical properties of the amino acids in the alignment. **(C-D)** AlphaFold (19) structural prediction of NUC-1 **(C)** and NUC-3 **(D)** with the basic helix-loop-helix domain as annotated by UniProt (16) highlighted in magenta. Colors in the predicted structures correspond to the AlphaFold per-residue confidence score (pLDDT). **(E)** Pairwise structural alignment of the structural prediction of NUC-1 and NUC-3 from the AlphaFold protein structure database (19) performed using the RCSB protein data bank (21). NUC-1 is indicated in gold and NUC-3 is indicated in blue.

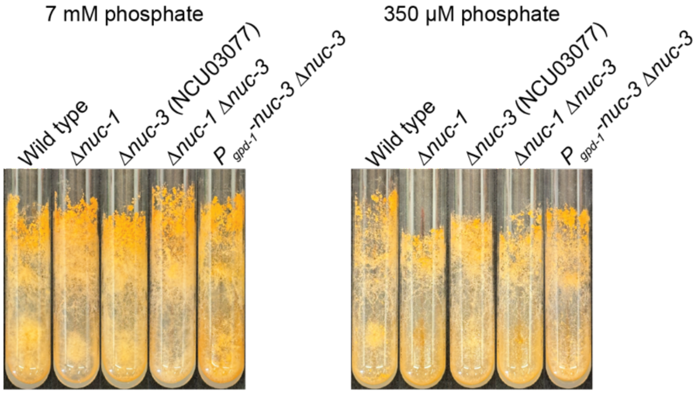

Fig S3. NUC-3 is not required for growth during phosphate limitation. The indicated strains were inoculated onto slants containing either sufficient phosphate (7 mM) or phosphate limitation (350 μM) (24) and grown for 2 d in constant dark and 6 d in constant light. Images are representative of three biological replicates.

SI TABLES

Table S1. Strains used in this study.

Strain name	Genotype	Source
Wild type	Wild type *mat A*	FGSC* 2489 (25)
Wild type	Wild type *mat a*	FGSC 4200 (25)
Δ*nuc-1*	Δ*nuc-1* (NCU09315)::*Hyg*^{R**} *mat a*	FGSC 11448 (26)
Δ*nuc-3*	Δ*nuc-3* (NCU03077)::*Hyg*^{R} *mat a*	FGSC 11356 (26)
Δ*nuc-3*	Δ*nuc-3::Nat*^{R***} *mat A*	This study (FGSC 26801)
Δ*nuc-1* Δ*nuc-3*	Δ*nuc-1::Hyg*^{R} Δ*nuc-3::Nat*^{R} *mat a*	This study (FGSC 26800)
$P_{gpd\text{-}1}$-*nuc-3* Δ*nuc-3*	Δ*csr-1::*$P_{gpd\text{-}1}$-*nuc-3* Δ*nuc-3::Nat*^{R} *mat A*	This study (FGSC 26802)

*FGSC stands for Fungal Genetics Stock Center (25).
**Hyg^{R} stands for the hygromycin resistance cassette (26).
***Nat^{R} stands for the nourseothricin resistance cassette (27).

Table S2. RNAseq library construction and sequencing.

Strain	Condition	Location	Sequencer	Read Length
Wild type *mat A*	7.3 mM phosphate (4 h)	Joint Genome Institute	HiSeq 2000	1x100 bp
Wild type *mat A*	0 mM phosphate (4 h)	Joint Genome Institute	HiSeq 2000	1x100 bp
Wild type *mat A*	0 mM phosphate (4 h)	UC Berkeley	HiSeq 4000	1x50 bp
Δ*nuc-1::Hyg*^{R} *mat a*	0 mM phosphate (4 h)	UC Berkeley	HiSeq 4000	1x50 bp
Δ*nuc-3::Hyg*^{R} *mat a*	0 mM phosphate (12 h)	UC Berkeley	NovaSeq	2x150 bp
Wild type *mat a*	0 mM phosphate (12 h)	UC Berkeley	NovaSeq	2x150 bp

Table S3. RT-qPCR primer sequences.

Gene	Forward primer 5' → 3'	Reverse primer 5' → 3'
act	TGATCTTACCGACTACCT	CAGAGCTTCTCCTTGATG
nuc-3	CGGGCCTAACACTGACTAGC	CCTATCCGGTTGCGTCTGTT
pho-2	TTTACGTTGACTCGTCGCCA	AGCGCCATATTCATACCGGG

SI REFERENCES

1. Vogel H. 1956. A convenient growth medium for *Neurospora* (medium N). Microbial Genetics Bulletin 13:42-43.
2. Fries N. 1938. Über die Bedeutung von Wuchsstoffen für das Wachstum verschiedener PilzeActa Universitatis Upsaliensis.
3. Beadle GW, Tatum EL. 1941. Genetic control of biochemical reactions in *Neurospora*. Proc Natl Acad Sci U S A 27:499-506.
4. Wu VW, Thieme N, Huberman LB, Dietschmann A, Kowbel DJ, Lee J, Calhoun S, Singan VR, Lipzen A, Xiong Y, Monti R, Blow MJ, O'Malley RC, Grigoriev IV, Benz JP, Glass NL. 2020. The regulatory and transcriptional landscape associated with carbon utilization in a filamentous fungus. Proc Natl Acad Sci U S A 117:6003-6013.
5. Galagan JE, Calvo SE, Borkovich KA, Selker EU, Read ND, Jaffe D, FitzHugh W, Ma LJ, Smirnov S, Purcell S, Rehman B, Elkins T, Engels R, Wang S, Nielsen CB, Butler J, Endrizzi M, Qui D, Ianakiev P, Bell-Pedersen D, Nelson MA, Werner-Washburne M, Selitrennikoff CP, Kinsey JA, Braun EL, Zelter A, Schulte U, Kothe GO, Jedd G, Mewes W, Staben C, Marcotte E, Greenberg D, Roy A, Foley K, Naylor J, Stange-Thomann N, Barrett R, Gnerre S, Kamal M, Kamvysselis M, Mauceli E, Bielke C, Rudd S, Frishman D, Krystofova S, Rasmussen C, Metzenberg RL, Perkins DD, Kroken S, et al. 2003. The genome sequence of the filamentous fungus *Neurospora crassa*. Nature 422:859-868.
6. Patro R, Duggal G, Love MI, Irizarry RA, Kingsford C. 2017. Salmon provides fast and bias-aware quantification of transcript expression. Nat Methods 14:417-419.
7. Love MI, Huber W, Anders S. 2014. Moderated estimation of fold change and dispersion for RNA-seq data with DESeq2. Genome Biol 15:550.
8. Langmead B, Salzberg SL. 2012. Fast gapped-read alignment with Bowtie 2. Nat Methods 9:357-359.
9. Zhang Y, Liu T, Meyer CA, Eeckhoute J, Johnson DS, Bernstein BE, Nusbaum C, Myers RM, Brown M, Li W, Liu XS. 2008. Model-based analysis of ChIP-Seq (MACS). Genome Biol 9:R137.
10. Bailey TL, Boden M, Buske FA, Frith M, Grant CE, Clementi L, Ren J, Li WW, Noble WS. 2009. MEME SUITE: tools for motif discovery and searching. Nucleic Acids Res 37:W202-W208.
11. Priebe S, Kreisel C, Horn F, Guthke R, Linde J. 2015. FungiFun2: a comprehensive online resource for systematic analysis of gene lists from fungal species. Bioinformatics 31:445-446.
12. Kanehisa M, Goto S. 2000. KEGG: Kyoto encyclopedia of genes and genomes. Nucleic Acids Res 28:27-30.
13. Stajich JE, Harris T, Brunk BP, Brestelli J, Fischer S, Harb OS, Kissinger JC, Li W, Nayak V, Pinney DF, Stoeckert CJ, Jr., Roos DS. 2012. FungiDB: an integrated functional genomics database for fungi. Nucleic Acids Res 40:D675-D681.
14. Katoh K, Standley DM. 2013. MAFFT multiple sequence alignment software version 7: improvements in performance and usability. Mol Biol Evol 30:772-780.

15. Price MN, Dehal PS, Arkin AP. 2010. FastTree 2--approximately maximum-likelihood trees for large alignments. PLoS One 5:e9490.
16. Consortium U. 2025. UniProt: the Universal Protein Knowledgebase in 2025. Nucleic Acids Res 53:D609-D617.
17. Huang X, Miller W. 1991. A time-efficient, linear-space local similarity algorithm. Adv Appl Math 12:337-357.
18. Madeira F, Madhusoodanan N, Lee J, Eusebi A, Niewielska A, Tivey ARN, Lopez R, Butcher S. 2024. The EMBL-EBI Job Dispatcher sequence analysis tools framework in 2024. Nucleic Acids Res 52:W521-W525.
19. Jumper J, Evans R, Pritzel A, Green T, Figurnov M, Ronneberger O, Tunyasuvunakool K, Bates R, Žídek A, Potapenko A, Bridgland A, Meyer C, Kohl SAA, Ballard AJ, Cowie A, Romera-Paredes B, Nikolov S, Jain R, Adler J, Back T, Petersen S, Reiman D, Clancy E, Zielinski M, Steinegger M, Pacholska M, Berghammer T, Bodenstein S, Silver D, Vinyals O, Senior AW, Kavukcuoglu K, Kohli P, Hassabis D. 2021. Highly accurate protein structure prediction with AlphaFold. Nature 596:583-589.
20. Varadi M, Bertoni D, Magana P, Paramval U, Pidruchna I, Radhakrishnan M, Tsenkov M, Nair S, Mirdita M, Yeo J, Kovalevskiy O, Tunyasuvunakool K, Laydon A, Žídek A, Tomlinson H, Hariharan D, Abrahamson J, Green T, Jumper J, Birney E, Steinegger M, Hassabis D, Velankar S. 2024. AlphaFold Protein Structure Database in 2024: providing structure coverage for over 214 million protein sequences. Nucleic Acids Res 52:D368-D375.
21. Bittrich S, Segura J, Duarte JM, Burley SK, Rose Y. 2024. RCSB protein Data Bank: exploring protein 3D similarities via comprehensive structural alignments. Bioinformatics 40:btae370.
22. Livingstone CD, Barton GJ. 1993. Protein sequence alignments: a strategy for the hierarchical analysis of residue conservation. Comput Appl Biosci 9:745-756.
23. Waterhouse AM, Procter JB, Martin DM, Clamp M, Barton GJ. 2009. Jalview Version 2--a multiple sequence alignment editor and analysis workbench. Bioinformatics 25:1189-1191.
24. Toh EA, Ishikawa T. 1971. Genetic control of the synthesis of repressible phosphatases in *Neurospora crassa*. Genetics 69:339-351.
25. McCluskey K, Wiest A, Plamann M. 2010. The Fungal Genetics Stock Center: a repository for 50 years of fungal genetics research. J Biosci 35:119-126.
26. Colot HV, Park G, Turner GE, Ringelberg C, Crew CM, Litvinkova L, Weiss RL, Borkovich KA, Dunlap JC. 2006. A high-throughput gene knockout procedure for *Neurospora* reveals functions for multiple transcription factors. Proc Natl Acad Sci U S A 103:10352-10357.
27. Kück U, Hoff B. 2006. Application of the nourseothricin acetyltransferase gene (*nat1*) as dominant marker for the transformation of filamentous fungi. Fungal Genetics Reports 53:9-11.
